# A Compartment-Based Mathematical Model for Studying Convective Aerosol Transport in Newborns Receiving Nebulized Drugs during Noninvasive Respiratory Support

**DOI:** 10.3390/pharmaceutics12100936

**Published:** 2020-09-30

**Authors:** Francesco Tarantini, Ilaria Milesi, Xabier Murgia, Federico Bianco, Raffaele L. Dellacà

**Affiliations:** 1 TechRes Lab, Dipartimento di Elettronica, Informazione e Bioingegneria (DEIB), Politecnico di Milano University, 20133 Milano, Italy; francesco.tarantini@polimi.it (F.T.); raffaele.dellaca@polimi.it (R.L.D.); 2 Department of Preclinical Pharmacology, R & D, Chiesi Farmaceutici S.p.A., 43122 Parma, Italy; f.bianco@chiesi.com; 3 Scientific Consultancy, 48640 Bilbao, Spain; xabi_murgia@hotmail.com

**Keywords:** aerosol neonates, noninvasive ventilation, mathematical model, lung deposition, convective transport

## Abstract

Nebulization could be a valuable solution to administer drugs to neonates receiving noninvasive respiratory support. Small and irregular tidal volumes and air leaks at the patient interface, which are specific characteristics of this patient population and are primarily responsible for the low doses delivered to the lung (D_DL_) found in this application, have not been thoroughly addressed in in vitro and in vivo studies for quantifying D_DL_. Therefore, we propose a compartment-based mathematical model able to describe convective aerosol transport mechanisms to complement the existing deposition models. Our model encompasses a mechanical ventilator, a nebulizer, and the patient; the model considers the gas flowing between compartments, including air leaks at the patient–ventilator interface. Aerosol particles are suspended in the gas flow and homogeneously distributed. The impact of breathing pattern variability, volume of the nebulizer, and leaks level on D_DL_ is assessed in representative conditions. The main finding of this study is that convective mechanisms associated to air leaks and breathing patterns with tidal volumes smaller than the nebulizer dramatically reduce the D_DL_ (up to 70%). This study provides a possible explanation to the inconsistent results of drug aerosolization in clinical studies and may provide guidance to improve nebulizer design and clinical procedures.

## 1. Introduction

Noninvasive respiratory support is nowadays the standard of care for spontaneously-breathing premature infants with respiratory distress (RDS) [1]. Therefore, the administration of pharmacological therapies by nebulization has been envisaged as the ideal combination to noninvasive respiratory support for targeting the immature lung [2]. Surfactant replacement therapy is a good example of such an approach: several clinical studies have attempted to deliver nebulized surfactant [3,4,5,6,7,8], although the efficacy of this delivery method remains inconclusive in part due to the lack of control of the surfactant aerosol dose delivered to the lung [9].

When a nebulizer is used in small newborns, the operational conditions of the set-up markedly differ from those commonly used in pediatric and adult patients: the newborn’s breathing pattern is characterized by very small tidal volumes and large temporal variability of both volumes and respiratory rate [10]. In this context, the assessment of the drug dose actually delivered to the patient is of primary interest, as clinical efficacy is correlated with the amount of the actual aerosol dose deposited in the lungs. Unfortunately, lung deposition and distribution measurements in premature infants would require invasive techniques and may not be warranted along with the treatment of these patients.

To circumvent this limitation, the most common approach for evaluating the delivery of aerosolized drugs relies on the estimation of the lung deposition from in vitro tests [11,12,13,14], in vivo preclinical studies [15,16,17,18], or mathematical modeling [19]. Most of these studies evaluated the overall drug deposition by quantifying the overall amount of aerosol that impacts against the walls of the different extra pulmonary components of the system (i.e., connecting tubes, patient interface, nebulizer chamber, and patients upper airways), the particles exhaled into the expiratory limb of the ventilation circuit, and the particles delivered into the lung [12,20,21]. These studies have been useful to investigate the impact of breathing flows, particle size, gravity, geometric characteristics of nebulizers, ventilation–patient interfaces and breathing circuits, and other fluid dynamic factors on the aerosol deposition.

Nevertheless, most of these studies are conducted using tightly sealed neonatal ventilation circuits and, therefore, they usually neglect the air leaks that take place during noninvasive ventilation at the patient interface. Air leaks may represent a significant aerosol loss during therapy in the neonatal intensive care unit; therefore, preclinical models that preclude air leaks at the patient interface may overestimate the lung dose. The most common interfaces used to provide ventilation support to patients are nasal prongs and nasal as well as facial masks [22]. Their use is often associated with relatively large air leaks [23], which result from the need of limiting the contact pressure of the interface with the fragile patient’s skin in order to avoid relevant injuries [24]. When nasal interfaces are used, leaks may also occur from the mouth even if neonates are mainly nasal breathers [25,26]. In these conditions, the relative contributions of the different mechanisms of aerosol transport towards the lungs can be markedly different to those typically encountered in pediatric or adult applications.

The aim of this study is to elucidate the role of the breathing pattern and leaks on the convective transport of the non-impacting aerosol fraction when nebulizers are used in premature infants with tidal volumes similar or even smaller than the volume of the nebulizer. To this aim, we developed a compartment-based mathematical model for determining how the convective transport of aerosol is affected by (a) the amplitude of tidal volume, (b) the breathing rate and relative duration of inspiration (respiratory duty cycle), (c) the variability of breathing pattern common in spontaneously-breathing premature neonates, and (d) the unavoidable air leaks at the patient interface typical of neonatal respiratory support setting.

## 2. Materials and Methods

We designed a mathematical model that encompasses a neonatal mechanical ventilator and a nebulizer connected to the patient by means of an interface (nasal prongs or mask) with the aim of analyzing the aerosol transport when a drug is nebulized in conjunction with noninvasive respiratory support.

**Figure 1:**
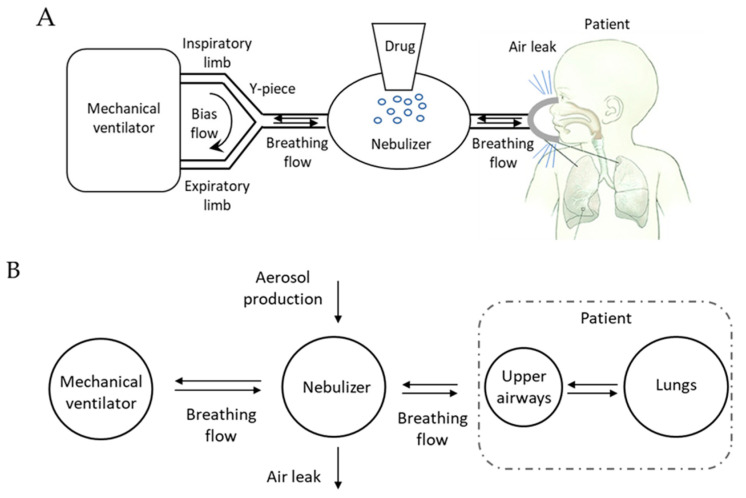
Architecture of the model. (**A**) Schematic representation of the set-up for neonatal applications during noninvasive respiratory support, where the nebulizer is placed between the Y-piece and the patient. The bias flow mentioned represents the flow of fresh breathing gas responsible for the maintenance of the pressure needed. (**B**) The compartmental model used to describe the system in which the compartment and flows are reported.

In neonatal settings, pressure support is commonly delivered to the baby by means of devices in which the pressure generator is embedded into the interface (e.g., variable flow CPAP generator). In this configuration, the nebulizer can only be placed along the inspiratory limb resulting in an overdiluted aerosol [27], which leads to very low lung deposition [11,12]. We are not considering that case but the most favorable, in terms of lung deposition: pressure support is generated by a two-limb mechanical ventilator and the nebulizer is connected between the Y-piece and the interface. In this configuration, the nebulizer is crossed only by the breathing flow of the baby, while the ventilation circuit is continuously flushed by a flow of fresh breathing gas (Figure 1A) used to generate the pressure. Even if not specifically needed for the sake of modeling, it is worthwhile to mention that only vibrating mesh nebulizer could be found at the Y-piece of ventilator [7].

The system was modeled as made of four compartments representing (1) the mechanical ventilator, (2) the nebulizers, (3) the patients’ conducting airways (i.e., upper airways and the intra-thoracic airways contributing to patient’s anatomical dead space), and (4) the patient’s lungs. The mass balance was used to evaluate aerosol transport within compartments, considering both the physical characteristics of each compartment and the flow developed by the patient during spontaneous breathing.

This model evaluates transport mechanism only, but provides a framework in which deposition models could be easily embedded, therefore the equations are derived by considering the following assumptions; (1) the aerosol moves exclusively as carried by convective flow, i.e., no diffusive mechanisms are considered; (2) there is no deposition of aerosol within the nebulizers or other conducting elements, such as patient interfaces or patient upper airways; (3) all aerosol entering the patient’s lung compartment during inspiration gets trapped into the lung, contributing to the Dose Delivered to the Lung (D_DL_); no aerosol particles are exhaled from the lung during expiration; (4) the nebulizers produces a constant amount of aerosol per time unit, which is injected in its chamber, independently of the amount of airflow passing through the device or of the amount of pressure applied to the patient; (5) aerosol particles reaching the Y-piece during exhalation are all washed out through the exhalation limb by the ventilator circuit bias flow; (6) air leaks are constant and independent from the breathing cycle, representing the administration of nasal Continuous Positive Airway Pressure (nCPAP) respiratory support. In the case of noninvasive ventilation, the higher pressure applied during inspiration is commonly associated to an increase of air leaks flow.

### 2.1. Model Compartments and Equations

Mechanical ventilator: This compartment produces the respiratory pressure support, provides fresh inspiratory gas free from aerosol on patient demand, and washes out all exhaled gasses and the aerosolized particles it contains. It is connected to the inlet of the nebulizers.

Nebulizer: This compartment represents a nebulizer which is modeled as made of a chamber with internal volume V_neb_ and an aerosol generator which produces a constant flow of aerosol (D_neb_). Gas volume in the chamber is assumed to be equal to the volume of the chamber and constant (no gas compression/storage is occurring), the amount of the total volume of the nebulized drug (q_neb_) is therefore neglected. Part of the aerosolized drug is not entering the patient because lost in the ambient by the unavoidable air leaks flow (${\dot{V}}_{\mathrm{leaks}}$) occurring at the connection between patient and nebulizer, introducing losses of nebulized drug. The nebulizer is exchanging gas flow with the ventilator (${\dot{V}}_{\mathrm{vent}}$) and the patient (${\dot{V}}_{\mathrm{patient}}$).

The equation determining the differential changes of the amount of aerosol within the nebulizer chamber during inspiration is, therefore,

(1) $${\dot{q}}_{\mathrm{neb}}\left(t\right)=D_{\mathrm{neb}}-{\dot{V}}_{\mathrm{patient}}\left(t\right)\left(\frac{q_{\mathrm{neb}}\left(t\right)}{V_{\mathrm{neb}}}\right)-\frac{q_{\mathrm{neb}}\left(t\right)}{V_{\mathrm{neb}}}{\dot{V}}_{\mathrm{leaks}}\left(t\right),$$

where q_neb_ represents the overall amount of aerosol within the device at a given time instant (t).

Similarly, the equation determining the differential changes in aerosol concentration within the chamber during patient expiration is

(2) $${\dot{q}}_{\mathrm{neb}}\left(t\right)=D_{\mathrm{neb}}+{\dot{V}}_{\mathrm{patient}}\left(t\right)\left(\frac{q_{\mathrm{uaw}}\left(t\right)}{V_{\mathrm{uaw}}}\right)-{\dot{V}}_{\mathrm{vent}}\left(t\right)\frac{q_{\mathrm{neb}}\left(t\right)}{V_{\mathrm{neb}}}-{\dot{V}}_{\mathrm{leaks}}\left(t\right)\frac{q_{\mathrm{neb}}\left(t\right)}{V_{\mathrm{neb}}},$$

where q_uaw_ is the amount of nebulized drug in the upper airways and V_uaw_ is the volume of that compartment.

The equation expressing the balance of mass which, as indicated above, neglects the contribution of the aerosol mass, is

(3) $${\dot{V}}_{\mathrm{patient}}\left(t\right)=-\left({\dot{V}}_{\mathrm{vent}}\left(t\right)+{\dot{V}}_{\mathrm{leaks}}\left(t\right)\right),$$

In all the equations of the model, positive values for airflow represent flows exiting from the compartment. All volume variables have been expressed in mL and times are in seconds.

Upper airways: this compartment represents the patient’s anatomical dead space, with a volume of V_uaw_. This compartment acts as a small reservoir for the nebulized drug and is crossed by the airflow determining the convective transport towards the patients’ lungs or backwards to the device compartment, according to the direction of the breathing flow ${\dot{V}}_{\mathrm{patient}}$.

Differential changes of the amount of aerosol within the upper airway compartment during inspiration are

(4) $${\dot{q}}_{\mathrm{uaw}}\left(t\right)={\dot{V}}_{\mathrm{patient}}\left(t\right)\left(\frac{q_{\mathrm{neb}}\left(t\right)}{V_{\mathrm{neb}}}-\frac{q_{\mathrm{uaw}}\left(t\right)}{V_{\mathrm{uaw}}}\right),$$

The equation expressing the mass balance for the aerosol concentration during patient expiration is

(5) $${\dot{q}}_{\mathrm{uaw}}\left(t\right)={\dot{V}}_{\mathrm{patient}}\left(t\right)\left(-\frac{q_{\mathrm{uaw}}\left(t\right)}{V_{\mathrm{uaw}}}\right),$$

Lungs: This compartment produces the breathing flow ${\dot{V}}_{\mathrm{patient}}$ responsible for transporting aerosol between the different compartments. The aerosol entering in this compartment during an inspiration is all trapped and contributes to the D_DL_. Therefore, the equation determining the differential changes of delivered dose is expressed for patient’s inspiration only:

(6) $${\dot{q}}_{\mathrm{delivered}}\left(t\right)={\dot{V}}_{\mathrm{patient}}\left(t\right)\left(\frac{q_{\mathrm{uaw}}\left(t\right)}{V_{\mathrm{uaw}}}\right),$$

The total %D_DL_ is defined as

(7) $$\%D_{\mathrm{DL}}=\frac{\int {\dot{q}}_{\mathrm{delivered}\;}\mathrm{dt}}{\int D_{\mathrm{neb}}\;\mathrm{dt}}\cdot 100,$$

### 2.2. Model Simulations

The dynamic of aerosol transport was simulated by implementing the model equations in Matlab^®^ V 2018a (Matworks Inc, Natick, MA, USA). The digital integration was done using the Euler method (discrete time interval of 10 ms), allowing the evaluation of the response of the system to any possible volume time series describing the breathing pattern of the subject.

This also allowed us to investigate the effects of different types of breathing patterns on %D_DL_.

Table 1 reports the list of all the simulations performed and the parameters associated.

The parameters space was explored to address for (1) breathing patterns variability (tidal volume (V_T_), respiratory rate (RR) and duty cycle that is expressed as inspiratory time divided by the total cycle time (T_i_/T_T_); (2) anatomy variability (V_uaw_); (3) nebulizer designs impacts (V_device_); and (4) airflow leaks (${\dot{V}}_{\mathrm{leaks}}$) level.

Simulation 1 accounts for the baseline scenario.

Regarding the volume time series, we run the simulations by feeding the model with both digitally created breathing waveforms resembling natural variability found in baby receiving noninvasive ventilation [28] (V_T_ = 2:20 mL, RR = 50:90 bpm, T_i_/T_T_ = 30%) and real volume traces recorded on neonates receiving respiratory support [29]. For the latter, a set of 10 real breathing signal of preterm newborns receiving CPAP was used (gestational age mean (SD) = 30^4^ (3^4^) weeks + day and weight mean (SD) = 1405 (606) g). Informed consent from parents of the infants who participated in the study was obtained before initiation of the study. The study was conducted in accordance with the Declaration of Helsinki and the protocol was approved by the Ethics Committee of A.O. San Gerardo–Monza-Italy (deliberation n°185, 23 July 2012).

Leakages were mathematically over-imposed on the volume waveform. Data were recorded in a previous study [29] using opto-electronic plethysmography [30] (OEP).

For the baseline scenario, the volume of the nebulizer was set equal to 10 mL (eFlow Neos, PARI Pharma, Starnberg, Germany); the volume of patient upper airways compartment was 1 mL, as extrapolated from anatomical models reported in literature [31]; and aerosol production estimates to 0.3 mL/min as extrapolated from literature [32].

All simulations were performed over a time period of 5 min, which allows the model to reach the equilibrium for all the simulations.

## 3. Results

**Figure 2:**
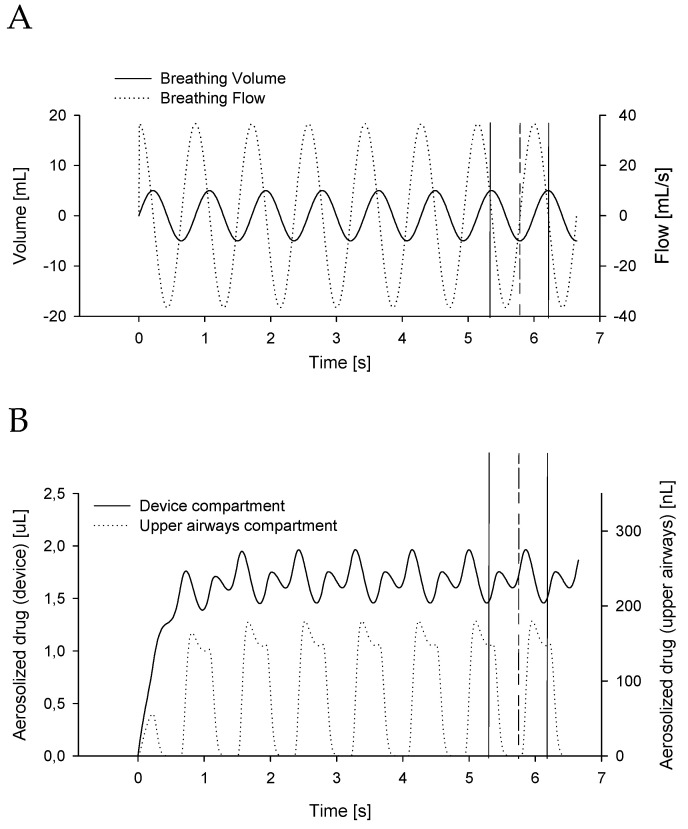
(**A**) Breathing volume and flow time courses fed to the model case (Table 1, Simulation 1). (**B**) Amount of aerosolized drug in the device compartment and the upper airways compartment. Vertical lines highlight, respectively, end inspiration, end expiration, and end inspiration again.

Figure 2 shows a representative time course of the main model variables generated by simulating the model at baseline conditions (Table 1, Simulation 1). A sinusoidal breathing pattern (volume and flow, Figure 2A) is fed to the model and the resulting aerosol concentrations both in the device and the upper airways compartments are reported over time in Figure 2B. A stable aerosol concentration in the device is reached after a few breaths. After stabilization, small oscillations of the concentrations in phase with patients’ breathing can be observed. As there is no aerosol production in the upper airways compartment and as its volume is significantly lower with respect to that of the device, the aerosol in this block is completely washed out during each expiration. This compartment is refilled with aerosol during the inspiratory phase, thus the aerosol concentration goes to a maximum in occurrences of the inspiratory flow peak of the subject.

**Figure 3:**
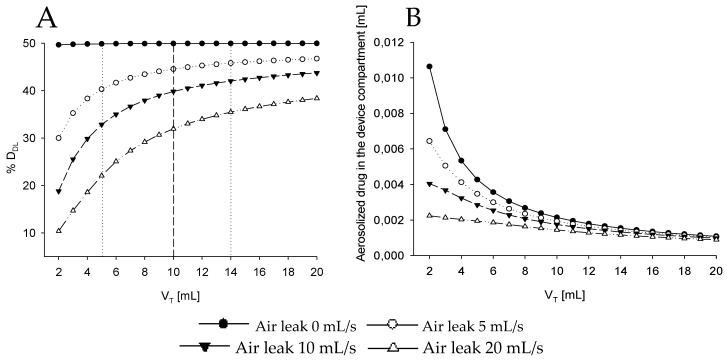
(**A**) Percentage of the drug delivered to the lung (%D_DL_) with respect to the total drug aerosolized over multiple tidal volumes (V_T_). (**B**) Averaged amount of aerosolized drug in the device compartment in case upper airways volume compartment has been neglected. Each line refers to a different air leak from 0 mL/s to 20 mL/s. Dotted lines at V_T_ equal to 5 and 14 mL highlight, respectively, the response of the system when two babies representing the upper and lower limit of the patient population are considered. The dashed line emphasized the response of the system at tidal volume smaller (left) or bigger (right) than the dead space of the nebulizer. V_T_ = tidal volume, D_DL_ = drug delivered to the lung.

In Figure 3, the estimated percentage of D_DL_ (%D_DL_) is reported at all V_T_ considering four different levels of air leaks: from 0 to 20 mL/s (Table 1, Simulation 2).

**Figure 4:**
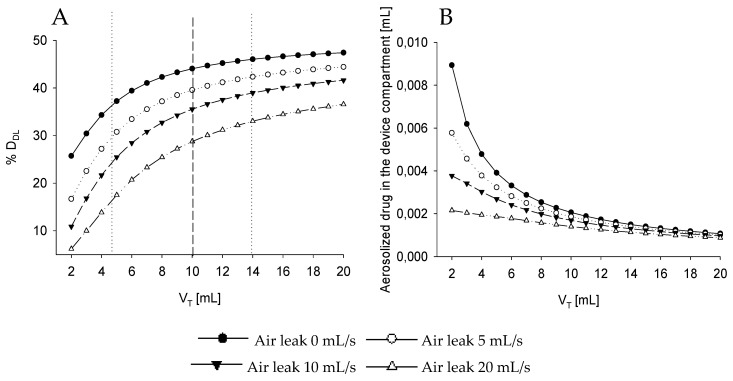
(**A**) Percentage of the drug delivered to the lung (%D_DL_) with respect to the total drug aerosolized over multiple tidal volume V_T_. (**B**) Averaged amount of aerosolized drug in the device compartment in case upper airways volume compartment has been sized at 1mL. Each line refers to a different air leak from 0 mL/s to 20 mL/s. Dotted lines at V_T_ equal to 5 and 14 mL highlight, respectively, the response of the system when two babies representing the upper and lower limit of the patient population are considered. The dashed line emphasizes the response of the system at tidal volume smaller (left) or bigger (right) than the dead space of the nebulizer. V_T_ = tidal volume, D_DL_ = dose delivered to the lung.

In this case, the volume of the upper airways has been artificially excluded (V_uaw_ equal to 0 mL), allowing the lungs compartment exchanging flow directly to the device compartment. In this way, the impact of this apparently small dead space could be better characterized by comparing results in Figure 4 in which it has been reintroduced.

In the absence of leakage, %D_DL_ is almost independent of V_T_ and close to 50% regardless of V_T_. As soon as leakages are introduced, %D_DL_ markedly reduces, especially when V_T_ is lower than V_neb_. For larger V_T_, the %D_DL_ at a given leak increases asymptotically towards a constant value that is strongly dependent on leakages, demonstrating how crucial leakages are on %D_DL_ at small V_T_ commonly found in newborns.

In Figure 3A two scenarios are also enlightened enclosing the variability in V_T_ expected for the population that could benefit from the aerosol treatment. The lower and upper limits of body weights of newborns of 26–33 weeks gestational age are 0.7 and 2.0 kg, respectively [33]. By considering a V_T_ of 6 mL/Kg, we may expect volumes ranging between 5 and 14 mL. In these specific cases, the %D_DL_ would always be greater than 35% regardless the amount of leakage for the biggest baby. Results are completely different for the smaller baby, where the leaks can reduce %D_DL_ down to 22%.

The average aerosol concentration in the device compartment increases for V_T_ smaller than the device compartment volume, due to the incomplete aerosol washout occurring at each breath, Figure 3B. As a consequence, the high concentration of aerosolized drug compensates for the reduced tidal volume in case of no air leaks granting for %D_DL_ close to 50%, but it also makes the system more sensitive to air leaks because the flow escaping from the interface will be more enriched of aerosol destinated to be wasted in the surrounding environment.

As a result, a greatly reduced air leak equal to 5mL/s reduces %D_DL_ from 50% to only approximately 45% in case V_T_ is larger than the nebulizer volume, but the same air leak can decrease %D_DL_ as low as 30% for V_T_ smaller than the device.

This behavior significantly changes when the upper airways compartment is added to the system (Figure 4, Table 1, Simulation 4). Even if the volume of this compartment is small (V_uaw_ = 1 mL) compared to the one of the nebulizer chamber (representing only 10% of V_neb_), it has a large influence on %D_DL_, which appears to be markedly reduced for all considered V_T_, especially for those lower than the volume of the chamber of the nebulizers. In particular, even in absence of leakage, the small asymmetry introduced by the upper airway dead space leads to a strong reduction in %D_DL_ which is largest at lowest V_T_ and reduces with increasing V_T_. The average aerosol amount in the nebulizers chamber shows similar trends to the one obtained without V_uaw_, but with lower averaged values.

Considering the same representative newborns mentioned above, the combined effect of leakage and V_T_ is even more emphasized, leading to a large reduction of %D_DL_ that gets to 33% for the large baby and 17% for small one when the 20mL/s leak is considered.

**Figure 5:**
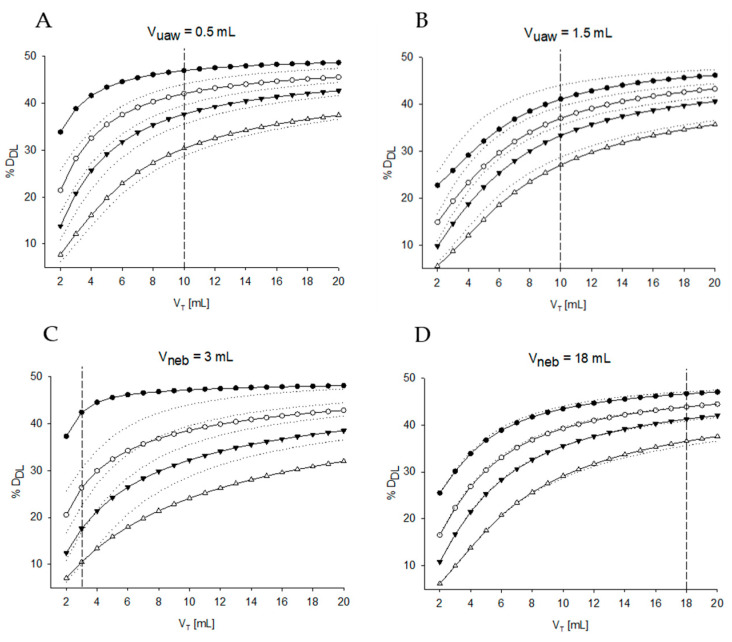
(**A**) Percentage of drug delivered to the lung (%D_DL_) over V_T_ in case the volume of the upper airways compartment is 0.5 mL or (**B**) 1.5 mL (Table 1, Simulation 4). (**C**) %D_DL_ over V_T_ in case the volume of the nebulizer is 3 mL or (**D**) 18 mL (Table 1, Simulation 5). Dotted lines represent the baseline condition (Table 1, Simulation 1). The dashed line represents the selected nebulizer volume and emphasizes the response of the system at tidal volume smaller (left) or bigger (right) than the dead space of the nebulizer. V_T_ = tidal volume, D_DL_ = dose delivered to the lung, V_neb_ = internal volume of the nebulizer, V_uaw_ = volume of the conductive airways.

The impacts of changing V_uaw_ and V_neb_ on %D_DL_ at the different V_T_ and leaks are reported in the simulations of Figure 5 (Table 1, Simulations 4 and 5). The figure shows that V_uaw_ is the parameter having the largest impact on %D_DL_, with V_neb_ having much lower effects.

**Figure 6:**
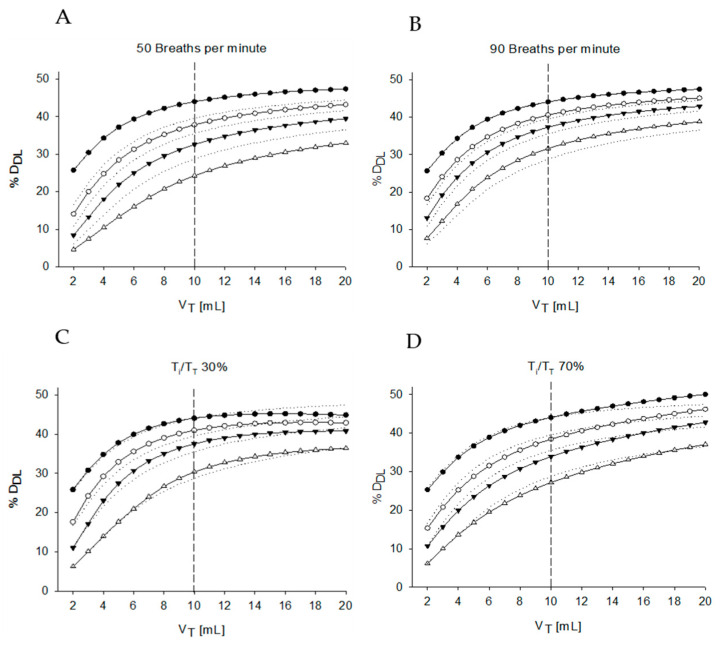
%D_DL_ over V_T_ evaluated varying one parameter at a time from the standard conditions: 50 breaths per minute (**A**), 90 breaths per minute (**B**), T_i_/T_TOT_ = 30% (**C**), and T_i_/T_TOT_ = 70% (**D**). V_T_ = tidal volume, D_DL_ = dose delivered to the lung, V_neb_ = internal volume of the nebulizer, V_uaw_ = volume of the conductive airways, T_i_/T_T_ = inspiratory time over total time.

In Figure 6 (Table 1, Simulations 6 and 7), the effects of different RRs and duty cycles are reported. RR does not markedly impact %D_DL_ when the system has no air leaks. However, an increase of RR impacts on %D_DL_ as much as the air leaks increased. This is due to the higher breathing flows entering the lungs compartment as the same V_T_ is delivered in a reduced time, which prevents the aerosol concentration in the nebulizers chamber to increase, and therefore the total amount of aerosol lost for a given leak flow is lower. Conversely, a decrease of RR reduces %D_DL_ which is showing a further decrease as the leakage flow increases, with larger variations compared to the ones observed when RR was increased.

Changing in T_i_/T_T_ is less relevant to the drug delivered to the lung. Nevertheless, the asymmetry hanging between the inspiratory and the expiratory phase results in changes in %D_DL_ which are largest at the highest V_T_ and when no leaks are present.

For all conditions tested, %D_DL_ is markedly reduced especially when the V_T_ of the patient becomes smaller than the volume of the chamber of the nebulizer, indicating that when a nebulizers is applied to newborns, the convective transport mechanisms lead to an unexpectedly large reduction in %D_DL_ with increased sensitivity to several parameters compared to what is expected in pediatric and adult settings.

**Figure 7:**
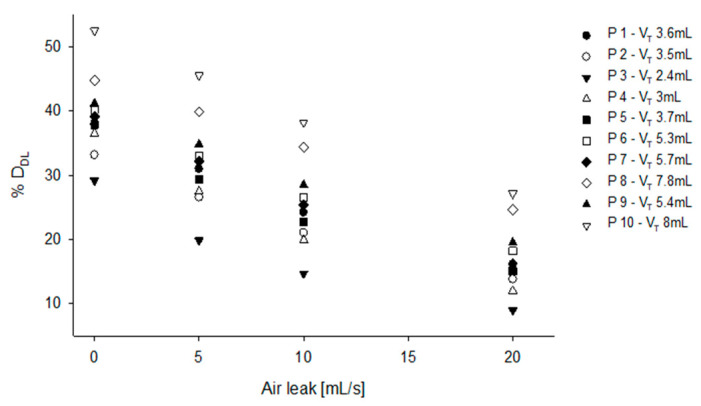
%D_DL_ obtained feeding the model with real breathing signals recorded on neonatal patients, varying the leak associated to each test.

When real volume traces (Figure 7, Table 1, simulation 8) recorded on patients are used for feeding the model, %D_DL_ ranged from 30% to 50% in case of no leak, and from 10% to 27% in case of 20 mL/s leak, showing that the irregular V_T_ and RR of the newborns further impact on %D_DL_.

## 4. Discussion

In this study, we developed a compartments mathematical model to evaluate the relative contribution of breathing pattern parameters, air leaks, the internal volume of the nebulizer chamber, and anatomical dead space on the aerosol convective transport, with special focus on the applications of nebulizers to small newborns receiving noninvasive respiratory support.

The main findings are as follows. (1) When the tidal volume becomes smaller than the nebulizer chamber, the insufficient washout leads to an accumulation of aerosol within the device. This increase in aerosol concentration has two main consequences: on the one hand it allows compensating the effects of the reduced convective transport by increasing the amount of aerosol entering the respiratory system per unit of gas volume but, on the other hand, it markedly increases the sensitivity of the amount of aerosol lost to other factors such as the unavoidable leaks between patients and the interface and the presence of an anatomical dead space between patient’s interface and the lung periphery. (2) Considering values for unavoidable leaks common in clinical practice and the physiological dead space of newborns, and by using actual breathing patterns recorded on patients, we found that the amount of aerosol entering the patients may range from 45% to as little as 15% of the total nebulized drug. These figures are obtained by considering only convective transport, i.e., without considering other relevant additional factors affecting deposition such as particles coalescence or impaction against the walls of the device, patient interface, or patient’s upper airways, as well as particles exiting the lung during exhalation. Considering the combination of all these factors, it appears extremely difficult to obtain accurate control of the actually delivered drug in clinical practice in this setting.

Some clinical studies have tried to investigate the possibility of nebulizing drugs in spontaneously breathing premature infants during nCPAP with inconclusive results [3,4,5,6,7,8]. These studies have shown that delivering nebulized medications to premature infants is a safe procedure, but only two studies have shown signs of therapeutic efficacy [3,7]. Minocchieri et al. have recently demonstrated that surfactant nebulized with a vibrating-membrane nebulizer reduces the need for intubation in 32^0^–33^6^ weeks^day^ gestational age (GA) infants with mild RDS (i.e., less premature infants), although no differences were observed in 29^0^–31^6^ weeks^day^ GA babies. One important limitation of clinical studies is the lack of information on the actual dose delivered to the lung, as all available quantitative tools are not suitable in such a fragile population. To overcome this limitation, lung deposition is often inferred from in vitro and in vivo studies. For instance, using a realistic neonatal nCPAP ventilation circuit composed of a neonatal ventilator, humidifier, an eFlow Neos vibrating-membrane nebulizer placed at the Y-piece, a cast of the upper airways of a premature infant (PrINT), and a breath simulator programmed with a sinusoidal neonatal breathing pattern, Bianco et al. determined that the lung dose of surfactant (poractant alfa) after nebulization accounted for 13.7 ± 4.0% of the nominal surfactant dose [21]. The authors reported that this intrapulmonary surfactant deposition sufficed to revert the respiratory distress in surfactant-depleted adult rabbits [21]. Similar lung deposition data were reported by Nord et al. in a scintigraphy study conducted with healthy piglets (1.2–2.2 kg) treated with the same drug/device combination [18]. In this study, mean lung deposition rates after nebulization during either nCPAP or nasal intermittent positive pressure ventilation (nIPPV) were 15.9% and 21.6%, respectively. A common limitation of both in vitro and in vivo models is the lack of air leaks at the patient interface. In vitro models are inspected and tightly sealed before conducting the experiments. Similarly, in vivo scintigraphy studies, the interface is tightly fitted in order to reduce the risk of exposure of the investigators to radioactive tracers, thus making it impossible to measure the effects of air leaks. These studies describe the lung dose under controlled conditions and therefore the information inferred from them may be regarded as the maximum achievable dose in a clinical setting. Taken together, there is no data describing the potential effect of V_T_ or air leaks on the dose delivered to the lungs in premature infants.

Our simulation data are in line with the experimental findings of previous in vitro and in vivo studies. For instance, Bianco et al. [12] found that the overall nebulized surfactant reaching the patient (i.e., adding extrapulmonary surfactant deposition to the amount reaching the lung) in a realistic in vitro neonatal noninvasive ventilation setting was approximately 50% when they used the settings and parameters of our baseline case reported in this study (Figure 2A).

However, our mathematical model suggests a striking synergic interaction of tidal volume and air leaks on the dose that can be potentially delivered to the lungs. When we introduced the impact of the leaks to our model, we found that the total amount of aerosol reaching the patient by convective transport is reduced from 50% to as little as 30% and this amount is further reduced to values even smaller than 20% when we also consider the impacts of lower tidal volumes, of the upper airway volume and of different breathing pattern timings. As these estimates are defined by considering the effects due to convective transport only, under the hypothesis that the fraction of aerosol depositing outside the patient’s lung (patient interface, upper airways, etc.) is independent of the total amount of aerosol, we can postulate that the overall lung deposition rate of ≈14% found by Bianco et al. can become as low as 5.6% in real clinical practice, potentially compromising the efficacy of the treatment.

As an additional finding, our model shows that not only leaks and breathing pattern can affect the overall aerosol transport, but they can also markedly increase the patient to patient variability of drug delivery. When real neonatal breathing tracings are used to simulate aerosol transport, we found that the aerosol effectively reaching the patient ranged to a minimum of 15% in patient P3 to a maximum of 40% in patient P10, when considering a constant leak of 10mL/s. If we apply the lung deposition rate from Bianco et al. again to this result, we get an overall lung deposition ranging from 4.2 to 11.2%, making it very difficult to predict the total amount of drug delivered to a given patient in these setting. Moreover, considering that leaks can also be highly variable during drug nebulization, we believe that our data may explain why the translation into clinical practice of nebulized surfactant did not show the expected efficacy, especially in small patients.

The amplified influence of leaks in neonatal applications, as highlighted by our model, suggests that, when the tidal volume of the patient is smaller than the volume of the device, it might be still possible to obtain high drug delivery rates but only if leaks are minimized. In this regard, it is important to avoid using interfaces that cannot provide a good patient–interface sealing. Therefore, we recommend to the clinicians to pay special care in obtaining the best possible fitting of the interface to the patient and to keep patients’ mouth closed. Moreover, the time of the procedure should be minimized and the patients’ gas exchange carefully monitored as the additional dead space with minimized leaks reduces actual minute ventilation to the lung. To reduce this critical dependency of the rate of drug delivery to leaks, the developers of nebulizers for small infants need to focus their efforts to design technical solutions able to minimize the internal volume of the device.

Our model also shows that small V_T_ are not increasing sensitivity of deposition rates to some parameters but are also reducing sensitivity to others. When the V_T_ of the patient is significantly larger than the internal volume of the nebulizer, all aerosol particles are removed from the device and inhaled by the patient during each inspiration. Conversely, during expiration, all aerosol is exhaled to the ambient, contributing to the loss of aerosolized drug. In this condition, changes in the T_i_/T_tot_ have a relevant direct influence on drug delivery [34]. However, when V_T_ is equal or smaller than the internal volume of the nebulizer, this wash-in/wash-out process cannot be completed and this leads to an increase of aerosol concentration within the devices at the beginning of the inspiration that partially compensates for the reduced T_i_, making the overall drug delivery much less sensitive to changes in duty cycle.

## 5. Limitations

As our goal was to specifically characterize the role of convective transport mechanisms in aerosol delivery to the patients, the model assumes that particles are all transported by the airflow independently of their size/mass and flow regimen. Therefore, all possible sources of aerosol loss/deposition outside the patient lung, such as particle coalescence, impacts with the walls of the device, interface or upper airways are not considered. All these factors are strongly dependent on the development of turbulent flow, therefore their relative contribution is expected to be dependent not only on the physical geometry of the components but also on the airflow speed, making this a potential additional factor impacting the relationship of aerosol deposition and tidal volume/respiratory time.

Compared to most modeling studies, we did not use computational fluid dynamic modeling (CFD). CFD modeling has the great advantages of being very accurate and also potentially able to characterize aerosol deposition due to impacts on the walls of the device, of the interface and the upper airways taking into consideration the development of turbulent flow and ducts geometry. However, CFD models require the detailed characterization of the geometry of all the components of the overall system and their results are function of a multitude of parameters and variables, making it more difficult to generalize the results and to identify general trends and relationships between variables of how the different parameters influence effectiveness of aerosol deposition. We therefore faced the problem by a different approach, i.e., by simplifying the problem by modeling only one of the aspects determining aerosol deposition for providing less accurate overall deposition results but more specific information on the factors affecting convective transport per se. We think that this approach can be more effective for an initial adjustment of the design of the devices and clinical procedures, leaving to CFD modeling the fine-tuning of future optimized devices and administration protocols.

This model is grounded on theoretical considerations. Therefore, even if the assumptions on which the convective transport modeling is based are relatively simple and straightforward, an in vitro validation should be performed in the future.

## 6. Conclusions

Our model suggests that in absence of air leaks, tidal volume alone does not represent a limiting factor on the dose of nebulized drug delivered to the lungs in neonatal applications. However, the combined effects of air leaks at the patient interface, which are constantly present in infants during nCPAP, and low tidal volumes can dramatically decrease the DDL. Clinical trials published so far enrolled infants ranging from 27 to 32 weeks gestational age (GA) with corresponding estimated birth weight from 700 g up to 2 kg, which accounts for a wide spectrum of tidal volumes. As a consequence, the results of such trials most likely describe the pharmacological effect of very different lung doses. In this regard, this novel data provides possible explanation to the apparently inconsistent results of clinical trials aimed to test the effectiveness of nebulized drugs in newborns. Moreover, our results may be used as starting point for additional research, for example providing input data for CFD models, allowing a more comprehensive and accurate description of aerosol deposition in neonatal applications and for providing guidance on how to improve nebulizer design and clinical procedures for developing more effective noninvasive drug delivery systems for newborns. 

## Figures and Tables

**Table 1:** List of the parameters set for each simulation.

Simulation	V_T_ [mL]	RR [bpm]	Ti/T_T_	V_neb_ [mL]	V_uaw_ [mL]	${\dot{V}}_{\mathrm{leaks}}\;[\mathrm{mL}/s]$
Simulation 1	10	70	50%	10	1	10
Simulation 2	2:20	70	50%	10	Not considered	0, 5, 10 20
Simulation 3	2:20	70	50%	10	1	0, 5, 10 20
Simulation 4	2:20	70	50%	10	0.5 (a) 1.5 (b)	0, 5, 10 20
Simulation 5	2:20	70	50%	3 (a) 18 (b)	1	0, 5, 10 20
Simulation 6	2:20	50 (a) 90 (b)	50%	10	1	0, 5, 10 20
Simulation 7	2:20	70	30% (a) 70% (b)	10	1	0, 5, 10 20
Simulation 8	Real parameters measured			10	1	0, 5, 10 20

V_T_ = tidal volume. Ti/T_T_ = inspiratory time divided total time, V_neb_ = internal volume of the nebulizer, V_uaw_ = volume of the conductive airways, ${\dot{V}}_{\mathrm{leaks}}$ = leak flow.
